# The Food and Drug Administration Biologics Effectiveness and Safety Initiative Facilitates Detection of Vaccine Administrations From Unstructured Data in Medical Records Through Natural Language Processing

**DOI:** 10.3389/fdgth.2021.777905

**Published:** 2021-12-22

**Authors:** Matthew Deady, Hussein Ezzeldin, Kerry Cook, Douglas Billings, Jeno Pizarro, Amalia A. Plotogea, Patrick Saunders-Hastings, Artur Belov, Barbee I. Whitaker, Steven A. Anderson

**Affiliations:** ^1^IBM, Washington, DC, United States; ^2^US Food and Drug Administration, Silver Spring, MD, United States; ^3^Gevity Consulting Inc., Vancouver, BC, Canada

**Keywords:** natural language processing, clinical notes, vaccine safety, vaccine adverse events, electronic health records

## Abstract

**Introduction:** The Food and Drug Administration Center for Biologics Evaluation and Research conducts post-market surveillance of biologic products to ensure their safety and effectiveness. Studies have found that common vaccine exposures may be missing from structured data elements of electronic health records (EHRs), instead being captured in clinical notes. This impacts monitoring of adverse events following immunizations (AEFIs). For example, COVID-19 vaccines have been regularly administered outside of traditional medical settings. We developed a natural language processing (NLP) algorithm to mine unstructured clinical notes for vaccinations not captured in structured EHR data.

**Methods:** A random sample of 1,000 influenza vaccine administrations, representing 995 unique patients, was extracted from a large U.S. EHR database. NLP techniques were used to detect administrations from the clinical notes in the training dataset [80% (*N* = 797) of patients]. The algorithm was applied to the validation dataset [20% (*N* = 198) of patients] to assess performance. Full medical charts for 28 randomly selected administration events in the validation dataset were reviewed by clinicians. The NLP algorithm was then applied across the entire dataset (*N* = 995) to quantify the number of additional events identified.

**Results:** A total of 3,199 administrations were identified in the structured data and clinical notes combined. Of these, 2,740 (85.7%) were identified in the structured data, while the NLP algorithm identified 1,183 (37.0%) administrations in clinical notes; 459 were not also captured in the structured data. This represents a 16.8% increase in the identification of vaccine administrations compared to using structured data alone. The validation of 28 vaccine administrations confirmed 27 (96.4%) as “definite” vaccine administrations; 18 (64.3%) had evidence of a vaccination event in the structured data, while 10 (35.7%) were found solely in the unstructured notes.

**Discussion:** We demonstrated the utility of an NLP algorithm to identify vaccine administrations not captured in structured EHR data. NLP techniques have the potential to improve detection of vaccine administrations not otherwise reported without increasing the analysis burden on physicians or practitioners. Future applications could include refining estimates of vaccine coverage and detecting other exposures, population characteristics, and outcomes not reliably captured in structured EHR data.

## Introduction

Vaccines are one of the most effective prevention tools available to support, promote and protect public health (1). One analysis estimated that routine childhood vaccinations prevented over 42,000 early deaths and 20 million cases of diseases in a cohort of children in the United States (U.S.) (2). Meanwhile, during the 2019–2020 influenza season, the Centers for Disease Control and Prevention (CDC) estimated that vaccination prevented an estimated 7.5 million influenza illnesses, 3.7 million influenza-associated medical visits, 105,000 influenza-associated hospitalizations, and 6,300 influenza-associated deaths (3). Vaccinations are also an effective tool for responding to pandemic events, and the accelerated timeline for vaccine research and development in response to COVID-19 has been a major success of the pandemic response effort (4).

Although vaccines are rigorously evaluated for safety prior to licensure, there is the possibility of adverse events following immunizations (AEFIs) occurring with exposure post-licensure in a larger general population, compared to limited exposure in the pre-licensure clinical trials. For instance, while pre-licensure vaccine trials may include up to several thousand patients, licensed vaccines are distributed and administered to a much larger cohort, at which time potentially rare AEs may be discovered (5–7). There may also be insufficient follow-up time in the clinical trial or the clinical study patients may not reflect the diversity and heterogeneity of the general population. Therefore, rigorous post-market vaccine safety surveillance studies that are powered to detect rare AEs are needed to address some of the limitations of randomized clinical trials (7, 8).

The U.S. Food and Drug Administration (FDA) Center for Biologics Evaluation and Research (CBER) is responsible for ensuring the safety, purity, potency, and effectiveness of biological products (9). This includes vaccines, allergenics, blood and blood products, as well as cells, tissues, and gene therapies for the prevention, diagnosis, and treatment of human diseases, conditions, or injury. To aid in this responsibility, CBER established the Biologics Effectiveness and Safety (BEST) Initiative with the aim of building data assets, analytics, and infrastructure for an active, large-scale, efficient post-market surveillance system for evaluating the safety and effectiveness of biologic products and developing innovative methods to support this initiative. Electronic health records (EHR), which have become nearly ubiquitous in U.S. clinical practice, have been leveraged to support post-market vaccine safety surveillance (7, 10–13). However, because EHRs are primarily developed for patient care and administrative purposes, their secondary use for research and surveillance can pose challenges (14). For instance, structured data elements—which are relatively easy to extract, store and analyze—have been leveraged by academics, practitioners, and regulators for surveillance and research purposes, while unstructured data—which includes a wealth of information found in patient clinical notes—remain relatively under-utilized because traditional analysis techniques are not able to easily extract information from the text contained in the clinical notes (14).

There are known issues associated with relying only on structured data to identify vaccine administration events, since some important vaccinations (e.g., influenza vaccines) are administered outside of EHR networks (e.g., in pharmacies, grocery stores, workplaces, and other settings) which often are not captured in the structured EHR data (15, 16). A 2014–2015 influenza season study found that non-medical settings accounted for 42.3% of influenza vaccinations among adults (16). This poses a challenge for detecting AEFIs given that many existing algorithms require evidence of a vaccine administration event in the form of structured EHR data. It is also a challenge for accurately monitoring regional or state level vaccination coverage—as relying only on EHR data could result in underestimates due to vaccines received outside of the EHR network—and accurate estimates are dependent on synthesizing data from multiple sources (e.g., EHR networks, state registries) (16).

Natural language processing (NLP) is one approach that can be used to improve the accessibility and usability of unstructured data found in EHRs (14, 17–23). NLP is situated at the intersection of computer science, artificial intelligence (AI), and linguistics, relying on machine learning techniques and rule-based algorithms to read, decipher, and make sense of (natural) human languages in order to make it of use to end-users (21, 22, 24). NLP includes the development of algorithms to automatically extract and structure information from free-text and/or semi-structured data sources (17, 18, 21, 22, 25). The application of NLP techniques for extracting information in biomedical data sources, such as EHRs, has been explored in a range of medical sub-specialties (18, 26–31). One notable study illustrated the feasibility of using NLP in a hospital EHR for pharmacovigilance purposes (28). The authors developed an NLP algorithm to identify medical outcomes which could be considered adverse drug events (ADEs) for seven different drugs/drug classes. Qualitative evaluation of the NLP algorithm suggested that previously unidentified ADEs could be detected in the EHR using the algorithm. The study provided a framework for the development of high-throughput and prospective systems, which could identify drug safety profiles throughout the entire market life of a therapeutic product (28). In a different study, an NLP algorithm was developed to identify local reactions associated with the tetanus diphtheria-acellular pertussis (Tdap) vaccine as reported in the Vaccine Safety Datalink database (31). The NLP algorithm achieved high accuracy, while demonstrating the potential of NLP to decrease the need for time-consuming and costly manual chart review and validation for vaccine safety studies (31).

Although there is research focused on the application of NLP techniques to improve the detection of outcomes (AEFIs and ADE), there is limited research focused on applying these techniques to improve the detection of vaccine administration data. Accurate vaccine administration data is important for monitoring regional vaccination coverage, and a necessary component to link vaccine exposure to an AEFI. This study describes the BEST Initiative's innovative and exploratory work developing an NLP algorithm to improve the accuracy of detection of vaccine exposure data in a large EHR database in the U.S.

## Methods

### Study Sample

The EHR data were sourced from an academic health system in Eastern United States with access to service data for over 5.4 million patients in the U.S. A population-level data characterization showed that, of the roughly 4 million patients seen from 2014 to 2019, nearly 500,000 received vaccine administrations, including 85,000 influenza vaccine administrations which were identified as such using this health system's proprietary codes. We chose this sampling approach under the assumption that the records of patients with coded evidence of vaccinations would be more likely to contain textual documentation of vaccinations than patients without coded documentation. We randomly sampled 1,000 influenza vaccine administrations (1.2% of total influenza vaccinations recorded in the EHR) and extracted all associated patient data for 2014–2019. This resulted in a dataset of 995 unique patients who have at least one, but maybe more, vaccinations in structured data. This dataset was used to develop and validate the NLP algorithm. The data were randomly divided into two datasets based on unique patients: a training dataset, which contained 80% (*N* = 797) of the patients, and a validation dataset, which contained the remaining 20% (*N* = 198) of the patients. The validation dataset was withheld from the NLP algorithm development process. A sample of vaccines detected by the algorithm in the validation data set was selected for clinical review to assess the performance of the algorithm. The goal of this study was to build a proof-of-concept algorithm that was highly accurate in detection vaccine administration in clinical notes. The validation set size was chosen to allow us to detect a PPV of ~90% (estimated PPV), with 95% Wilson confidence intervals (95% CIs) range of max 10% around the PPV estimate. Due to the study's small sample size and to avoid overshoot, where the commonly used Wald CI would exceed possible values (i.e., >100% PPV), the PPV confidence interval will be calculated using Wilson method (32).

### Algorithm Development and Clinical Review

All analyses were developed and implemented using custom Python scripts (33). Standard NLP techniques were used to mine unstructured data (24, 34). Briefly, clinical notes from the training dataset were processed and fed into an NLP rule-based matching algorithm, to identify evidence of vaccine administration. Two clinicians served as subject matter experts (SMEs) during the development and validation of the NLP algorithm, guiding the selection of the keywords and vaccine type term sets for the rule-based matching algorithm, supporting the iterative manual review of cases in the training dataset to improve the algorithm performance, and verifying vaccine administration in patients' medical charts. The clinician reviews of the patients' medical charts including both structured and unstructured data served as the gold standard for this study to assess whether the NLP algorithm was successfully identifying patients that had a vaccine administered. Clinical verification of vaccine administration was blinded. Table 1 summarizes the steps taken to create the NLP algorithm.

**Table 1:** Vaccine administration evidence algorithm steps.

**Step**	**Description**
1. Filter unstructured notes by type	Medical notes are often characterized by their type (e.g., Discharge Summary, Surgical History). In this case, certain note types were filtered out because the study team judged certain note types to have a reduced probability of containing free-text documentation of vaccinations based on manual review of a sample of our training set. Examples include notes populated with semi-structured interview questions like “Received Flu Vaccine: No” or patient education notes that discuss vaccinations in the hypothetical and might read “...after this procedure, do not receive a flu shot for at least a month...”.
2. Tokenize Filtered Set of Notes	In order to process the filtered set of notes, we used a simple tokenize algorithm (SpaCy)^1^, to segment the text from the filtered notes into single words.
3. Create simple part of speech tagging to identify presence of vaccine administration	Using the list of words produced by the tokenizer algorithm, we tagged verbs which indicated vaccine administration. The identification of a past tense verb (“got,” “received,” “given,” or “had)” assisted in identifying true instances of vaccination rather than vaccine education materials (Full list can be found in Supplementary Material)
4. Using NLP rule-based matching to search for vaccine derivative in vicinity of verb	If a desired verb was found, the algorithm searched for evidence of a vaccination (e.g., vaccine, shot, vaccination) within five tokens, where a token is a continuous string of characters between a space or punctuation marks.
5. Using NLP rule-based matching to search for and identify vaccine type	The algorithm used the preceding four tokens of the vaccination term to search for the vaccine type (e.g., influenza, flu, hep b, hepatitis b). It then looked for the mapped term that is the most complete match of the four preceding tokens (e.g., “pneumococcal 13” maps to “pneumococcal 13-valent” rather than more generic “pneumococcal)”. The table of the mappings to vaccine types was developed from an initial list from clinicians SMEs augmented by potential alternative names found in a manual review of a sample of training cases. The final table can be reviewed in Supplementary Material. If a name was not found, the vaccine was added as “vaccine” with no specified type (e.g., if note read “patient received vaccinations today)”.
6. Find or derive date of vaccine administration	The algorithm searched for an absolute date (ex. 1/12/19, 1/19) or relative date (yesterday, last week, today) within five tokens of the vaccination term. A table of the different date formats and relative date tokens used can be found in Supplementary Material. We built the list of date formats and selected five tokens as the window from the vaccination term based on a developer's manual review of a sample of cases from our training dataset. Relative dates were derived based on the date of the note entry. Vaccinations were only included when an associated absolute or relative date was found. Manual reviews demonstrated that, without absolute or relative dates, mentions of vaccinations were much less likely to represent actual vaccination events. As this was a POC algorithm, the algorithm is limited in the permutations of date formats it can identify and could be improved by the ability to recognize phrases like “3 days ago” or “3 weeks ago” among others.

*1https://spacy.io/usage/processing-pipelines*

**Figure 1:**
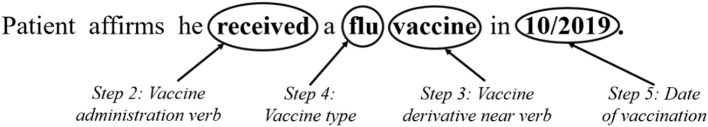
NLP algorithm example, steps 2 through five.

Figure 1 illustrates a hypothetical example of a vaccine administration note, with corresponding time and date, detected by the NLP algorithm created via the process described in Table 1. This figure presents the process after the notes are filtered by type (i.e., Step 1 is complete). The past tense verb of “received” was identified to indicate vaccine administration. With the identification of this verb, the algorithm then searched for a vaccine-derivative (e.g., vaccine, shot) within five tokens. The algorithm then identifies “vaccine” within five tokens of “received.” The number of tokens was selected upon manual review and assessment of the performance of various counts of tokens. The next step in the algorithm is to identify the vaccine type by searching the four tokens preceding the vaccine-derivative, which in this case is “flu.” See Table 1: Step 5 for a more detailed description of our approach to matching vaccine types and dealing with the lack of a standardized name for a reported vaccine administration. Finally, the algorithm searches for an administration date, either absolute or relative. The example presented in the figure is an absolute date of 10/2019. We tested existing date-expression mappers for this purpose but found that they did not perform well on some of the notes so created our own Regex formula to detect dates in the patient records.

Each NLP detected vaccination event was linked to a known administration event in the structured data to identify those that were successfully captured in the structured data and those that were not. To conduct the match, we first searched for vaccine administrations a month before or after the derived date flagged by the NLP algorithm. Of the administrations found within a month, we compared the name of the vaccine administration with the standardized name of the vaccine. The cut-off of 1 month was selected as an inclusive interval to allow matches when patients had slightly inaccurate recall of an earlier vaccination and its date. If the standardized name was contained within the record name, we considered it a match (e.g., Standardized Name: “Influenza,” Record Name “Influenza, inactivated virus”-see Supplementary Material for more information).

### Algorithm Validation/Performance Assessment

The final NLP algorithm was applied to the validation dataset (*N* = 198). Full medical charts for 28 vaccine administrations detected using the NLP algorithm—selected randomly from among the convenience sample of flu-vaccinated individuals—were provided to and reviewed by the clinical SMEs. Review was stopped after 28 vaccines were reviewed because we were already able to show that within a 95% Wilson CI, our algorithm had at least >80% PPV which we felt was adequate to demonstrate that this POC algorithm showed promise. For additional research on this algorithm, we would suggest reviewing additional cases to decrease the confidence interval range and obtain a more specific estimate of PPV. For the validation process, a semi-automated chart review tool was developed to reduce the time needed to conduct chart reviews and to improve the accuracy of the abstracted data. The tool populates relevant patient data into a centralized location and includes a timeline view and enhanced clinical note search functionality, allowing for rapid review of cases by clinicians. Clinicians were instructed to review charts to confirm a vaccine exposure but were blind to which cases had no structured vaccine administration data, which vaccine was administered and whether the NLP algorithm detected a vaccine exposure or not.

Upon reviewing the structured and unstructured chart data, the clinicians determined the likelihood that a true vaccine administration with the correct accompanying date was identified. The likelihood was categorized using the following Likert scale: Definite, Probable, Possible, Doubtful, Ruled Out, Not Determined (35). This scale was adapted from the Adverse Drug Reaction (ADR) Probability Scale to suit the context of this work. The results of review of the 28 vaccine administrations selected from our validation set were used to estimate the positive predictive value (PPV) of the algorithm. The clinicians reviewed all cases independently, and no conflicts arose.

Finally, in order to understand how the algorithm might help expand detection of vaccinations, after validation of the NLP algorithm, the NLP algorithm was applied to the entire sample of 995 patients, in order to quantify vaccine administrations that were not recorded in the structured EHR data elements. Vaccines identified by the NLP algorithm were linked programmatically to vaccines identified in the structured data using the relevant logic, based on vaccine type and administration date reported. The percent change in vaccine administration identified using the NLP algorithm compared to using structured vaccination data alone will be calculated using the formula below:


(1) $$\begin{array}{l} \frac{\begin{array}{c} N\;Vaccine\;Administrations\;Detected\\ by\;NLP\;algorithm\;in\;Clinical\;Notes \end{array}}{\begin{array}{c} N\;Vaccine\;Administrations\;Captured\\ in\;Structured\;Vaccination\;Records \end{array}}\times \;100 \end{array}$$

## Results

### Sample Population

Table 2 presents details of the 995 patients selected for the development of the algorithm, as well as details about their vaccine administrations. The majority (54.3%) of patients were 18–65 years of age, most (54.6%) were female, and a plurality (45.9%) were of Caucasian/white race/ethnicity. This was similar to the distribution in the EHR dataset overall. Among the 995 patients, there were 2,740 vaccine administrations identified in the structured data, of which 1,706 (62.3%) were influenza vaccinations.

**Table 2:** Characteristics of the sample population and broader EHR population, 2014–2019.

	**Sample distribution**	**EHR distribution**
Patient characteristic	*N* = 995	*N* = 6,831,127
Age (median, IQR)	51.37 (26–63)	NA
**Age-group (Per cent, %)**		
<18	19.70	10.14
18–65	54.27	64.63
65+	22.61	25.21
Unknown	3.42	0.03
**Sex (Per cent, %)**		
Male	45.63	41.51
Female	54.27	58.42
Other/unknown	0.10	0.07
**Race (Per cent, %)**		
Asian/Pacific	2.11	1.77
Black/African American	41.61	34.48
Caucasian/White	45.93	42.14
Other	10.25	21.61
**Structured vaccination data (** ***n*** **)**		
Vaccine administrations	2,740	NA
Influenza vaccine administrations	1,706	NA
Medical encounters	2,068	NA

*Age was calculated from the start of the study period (January 1, 2014). Patients born within the study period were automatically added to the <18 group.*

*NA, Not available*.

### Validation

Among the 28 vaccine administrations reviewed by clinicians, 27 were determined to be “definite” while one was “possible” (*data not shown*). This would equate to a PPV of 96.4% with a Wilson 95% Confidence Interval range of 82.3% to 99.4% associated with the NLP algorithm. A total of 18 (64.3%) had linked evidence to a vaccination event in the structured vaccination data, while 10 (35.7%) were found solely in the unstructured clinical notes.

### Full Sample Results

**Figure 2:**
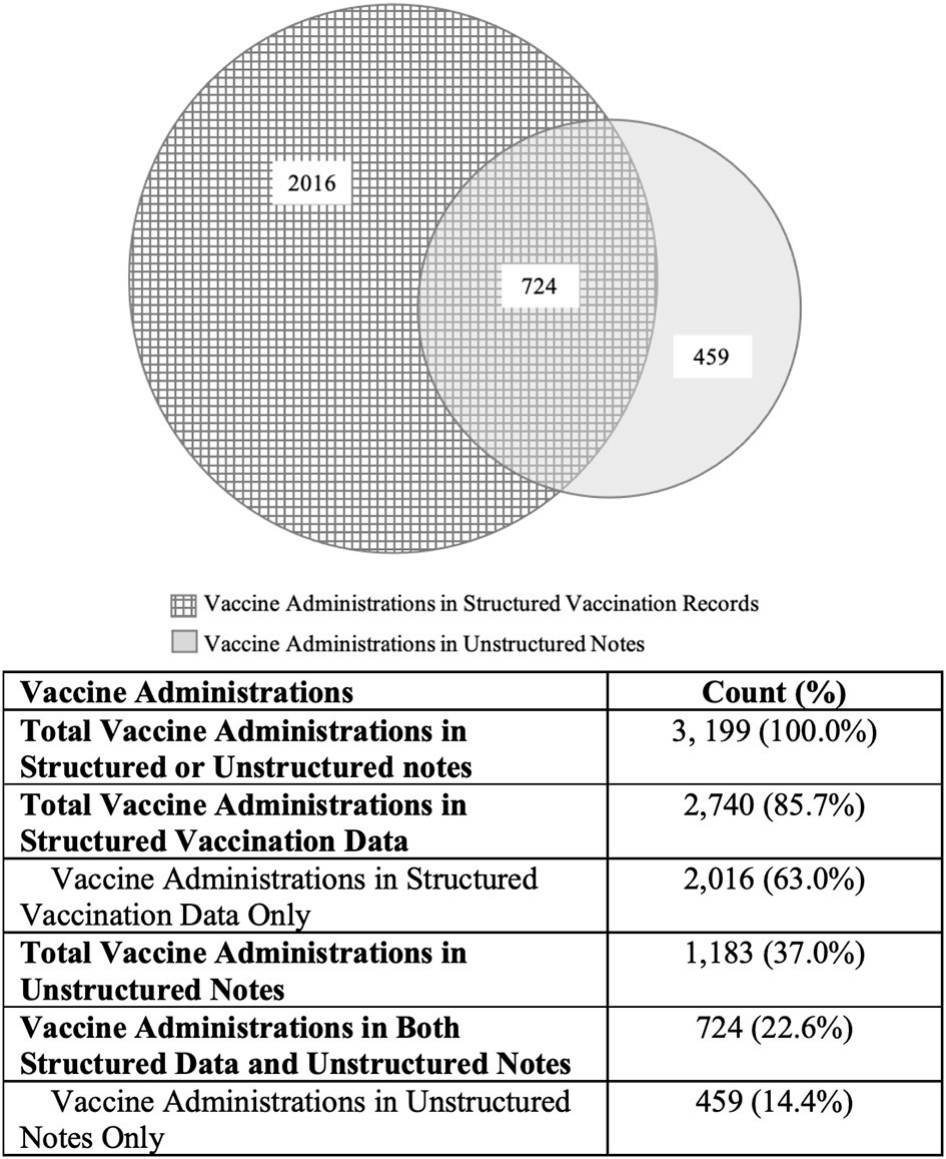
Vaccine administrations identified in structured vaccination data and unstructured notes.

Figure 2 illustrates the vaccine administrations identified in the structured data and unstructured notes. Among 995 patients, a total of 3,199 vaccine administrations were identified in the structured vaccination data and the unstructured clinical notes.

The NLP algorithm detected 1,183 vaccinations (37.0%), of which 724 (22.6%) were also reported in the structured data. The remaining 459 (14.4%) were captured in the clinical notes only. This represents a 16.8% increase in vaccine administration identification compared to using structured vaccination data alone, as calculated below:


(2) $$\begin{array}{l} \frac{\begin{array}{c} 459\;Vaccine\;Administrations\;Detected\\ by\;NLP\;algorithm\;in\;Clinical\;Notes \end{array}}{\begin{array}{c} 2,740\;Vaccine\;Administrations\;Captured\\ in\;Structured\;Vaccination\;Records \end{array}}\times \;100=16.8\% \end{array}$$

Among the 459 vaccines captured only in the clinical notes, 129 (28.1%) were influenza vaccinations.

## Discussion

The application of rule-based NLP techniques to extract information from unstructured data in biomedical sources, including EHRs, has the potential to improve both surveillance and research (14, 17–20, 20–23). In the present analysis, we developed and applied a simple NLP algorithm to extract vaccine administration data from the clinical notes in a large academic health system's EHR. The NLP algorithm identified an additional 459 vaccine administrations (complete with type and date) compared to structured data alone, resulting in a 16.8% increase in exposure identification of all types of vaccine administration we search for, including both flu and other types of vaccines. This demonstrates that our algorithm can accurately identify vaccine exposures from the unstructured data, including ones captured in the structured data and those that would otherwise go unidentified.

Furthermore, a rapid validation exercise of 28 NLP-identified vaccine administration events produced a PPV of 96.4% (Wilson 95% CI range: 82.3–99.4%), validating the utility of the NLP algorithm to accurately detect vaccine administrations in the unstructured clinical data with minimal false positives. Most studies applying NLP techniques to immunization data have focused on the detection of outcomes related to AEFIs. However, identification of a vaccine administration event is a necessary step in the AEFI detection process, and past studies have suggested that a substantial proportion of certain vaccine administrations (e.g., 42.3% of adult influenza vaccinations during the 2014–2015 influenza season) occur outside of medical settings (16). These administrations would not be captured in the structured EHR data, suggesting that improving exposure detection capabilities would contribute to enhancing AEFI surveillance and monitoring. Specifically, leveraging an NLP algorithm could support the detection of a proportion of vaccinations received outside the health provider's EHR, but which have been reported in the unstructured clinical notes, which can help increase reporting of adverse events in cases where the patient receives care in a different location than they received their vaccination. NLP data extraction techniques could also be used to supplement traditional vaccination coverage surveillance methods using structured data in order to address vaccine administration data gaps among those who receive vaccines outside EHR coverage areas (15, 16, 36).

Given that the developed NLP algorithm is not specific to vaccine type, it can be easily adapted with minimal modification to other vaccines and EHR systems to improve the quality of vaccine data and mitigate the effect of missing data in EHRs due to vaccine administrations outside of the EHR network (i.e., in non-medical settings). For instance, for those who have a documented influenza vaccine, the algorithm could be adapted to detect severe acute respiratory syndrome coronavirus 2 (SARS-CoV-2) vaccine administrations from clinical notes. SARS-CoV-2, which causes COVID-19, may result in a disease presentation similar to that of influenza. The work presented here may be particularly applicable to COVID-19 vaccine identification for safety and effectiveness studies, administration of COVID-19 vaccines outside traditional medical settings may be particularly common and fee-for-service records such as administrative claims data may not be available in a timely manner.

This study was designed as a proof-of-concept to determine if rule-based NLP techniques can be applied to enhance identification of vaccination exposure data to those with a previously recorded vaccination in EHR databases. While we demonstrated the utility of using NLP techniques to identify additional vaccination events not available in structured data alone, further study of its application in distinct EHR systems and with larger samples would help quantify the extent of the benefit. Other limitations include resource constraints, which resulted in the validation of a small sample of the NLP-identified vaccination events, and the estimated PPV may not be generalizable to other EHR systems. A full external validity study is required to assess performance in additional detail. Further, our approach of identifying and assessing a cohort of flu-vaccinated patients (as identified in the structured data) may have reduced the generalizability of our findings, as the selected records may have been more likely to contain vaccine information. We also did not set out to provide an estimate of the extent of missingness in structured EHR data, and the algorithm performance assessment did not include metrics such as sensitivity, specificity, and negative predictive value; this is because the main goal was to create a precise algorithm with minimal false-positives, even if the NLP pipeline missed some legitimate cases that would have otherwise been identified in the structured notes. We did not review all cases in the test set to enable calculation of these metrics, though recognize that this could be an avenue for further study.

Other avenues for future research include enhancements to the algorithm to better capture common abbreviations and misspellings relevant to vaccine administrations and other date expressions not currently included in the NLP algorithm (e.g., “vaccine administration X days ago”). Additional enhancement to identify the setting in which each vaccine administration occurred would also be of value to determine whether vaccines were administered in a medical or non-medical setting. Adaptations for use with other vaccine types, such as COVID-19, would also be of value. The NLP techniques could also be leveraged to improve data reported to Vaccine Adverse Event Reporting System (VAERS) by allowing participating clinics to identify both vaccinations events and AEFIs more accurately. Underreporting of AEFIs to VAERS may hinder identification of AEFIs associated with FDA-licensed vaccines, and this effort would support a long-term goal of the BEST Initiative to improve the post-licensure vaccine safety monitoring system used by FDA and CDC (37).

## Data Availability Statement

The data analyzed in this study is subject to the following licenses/restrictions: due to the nature of this research, participants of this study did not agree for their data to be shared publicly, so supporting data is not available.

## Ethics Statement

Ethical review and approval was not required for the study on human participants in accordance with the local legislation and institutional requirements. Written informed consent from the participants' legal guardian/next of kin was not required to participate in this study in accordance with the national legislation and the institutional requirements.

## Author Contributions

MD: methodology, software, validation, formal analysis, data curation, writing-original draft, visualization, and supervision. HE: methodology, writing- review and editing, and supervision. KC: software, validation, formal analysis, writing-original draft, and visualization. DB: methodology, software, formal analysis, and data curation. JP: methodology, software, writing-original draft, and supervision. AP and PS-H: writing- review, and editing. AB and BW: methodology, writing- review and editing. SA: funding acquisition, methodology, and supervision. All authors contributed to the article and approved the submitted version.

## Funding

This project was funded through the U.S. Food and Drug Administration Center for Biologics Evaluation and Research Biologics Effectiveness and Safety (BEST) Initiative. Several co-authors hold commercial affiliations with IBM and Gevity Consulting Inc. IBM provided support in the form of salaries for authors (MD, KC, DB, JP), but did not have any additional role in the study design, data collection and analysis, decision to publish, or preparation of the manuscript. Gevity Consulting Inc. provided support in the form of salaries for authors (AP, PS-H) but did not have any additional role in the study design, data collection and analysis, decision to publish, or preparation of the manuscript.

## Conflict of Interest

MD, KC, DB, and JP are employed by IBM. AP and PS-H are employed by Gevity Consulting Inc. The remaining authors declare that the research was conducted in the absence of any commercial or financial relationships that could be construed as a potential conflict of interest.

## Publisher's Note

All claims expressed in this article are solely those of the authors and do not necessarily represent those of their affiliated organizations, or those of the publisher, the editors and the reviewers. Any product that may be evaluated in this article, or claim that may be made by its manufacturer, is not guaranteed or endorsed by the publisher.
