# Supplementary material for: The Food and Drug Administration Biologics Effectiveness and Safety Initiative Facilitates Detection of Vaccine Administrations From Unstructured Data in Medical Records Through Natural Language Processing
Source: Front Digit Health. 2021 Dec 22;3:777905. doi: 10.3389/fdgth.2021.777905 (PMC8727347; doi:10.3389/fdgth.2021.777905)
Supplement: Supplementary file 1 [file Table_1.docx]

Supplementary Material

**Appendix**

**Table S1.** Administration Verbs and Vaccine Term-sets for the NLP algorithm

| **Type** | **Terms** |
| --- | --- |
| Administration Verb | 'got', 'received', 'given', 'had', 'administered' |
| Vaccine Derivative | 'vaccine', 'vaccines', 'vaccination', 'vaccinations', 'shot', 'shots', 'immunization', 'immunizations' |
| Influenza Vaccine | 'flu', 'flulaval', 'influenza', 'influenza virus', ‘fluad’, ‘live attenuated influenza’, 'lai', 'laiv', ‘influenza, inactivated virus’ |
| Measles/Mumps/Rubella | 'measles/mumps/rubella', 'measles/mumps/rubella virus', 'mmr II',  'mmr' |
| Hepatitis | ‘hepatitis a adult’, ‘hep b’, ‘hepatitis a’, ‘hepatitis b’, 'hepatitis a pediatric', 'hepatitis b pediatric' |
| Haemophilus | ‘haemophilus’, ‘hib’, 'haemophilus b conj (PRP-OMP)' |
| Meningococcal | 'meningococcal conjugate', 'meningococcal', 'meningococcal polysaccharide', 'meningococcal group b’, 'meningococcal polysaccharide' |
| Pneumococcal | 'pneumonia', 'pneumococcal' |
| Pneumococcal 13-valent conjugate | 'pneumococcal 13-valent', 'PCV 13' |
| Pneumococcal 23-valent | 'pneumococcal 23-valent' |
| Pneumococcal 7-valent | 'pneumococcal 7-valent' |
| Poliovirus | ‘poliovirus’ |
| Rotavirus | ‘rotavirus’ |
| Tetanus | ‘tetanus’ |
| Typhoid | ‘typhoid’ |
| Varicella | ‘varicella’ |
| Zoster | ‘zoster’ |
| Tetanus, Diphtheria, Pertussis | ‘tdap’, ‘dtap’ |
| Human Papillomavirus | ‘human papillomavirus’ |

**Table S2.** Date Formats

| **Absolute Date Format** |
| --- |
| MM/YYYY |
| M/YY |
| MM/DD/YYYY |
| M/DD/YYYY |
| M/D/YYYY |
| M/D/YY |
| MM/D/YYYY |
| MM/D/YYYY |
| **Relative Date Terms** |
| today, yesterday, afternoon, morning, am, pm, Monday, Tuesday, Wednesday, Thursday, Friday, Saturday, Sunday |
